# Reasonable water and fertilizer ratios regulate fruit cell tissue structure and improve the quality of fragrant pear in China’s extreme arid regions

**DOI:** 10.3389/fpls.2026.1845035

**Published:** 2026-07-02

**Authors:** Tianle Li, Qiangqing Zheng, Qiling Chen, Shijie An, Zhihui Tang, Xinlu Bai, Jinhu Zhi

**Affiliations:** 1 College of Agriculture, Tarim University, Alar, China; 2 Tiemenguan Experimental Station, Xinjiang Academy of Agricultural Reclamation Sciences, Tiemenguan, China; 3 Institute of Forestry and Garden, Xinjiang Academy of Agricultural Reclamation Sciences, Shihezi, China; 4 Research Center of Oasis Agricultural Resources and Environment in Southern Xinjiang, Tarim University, Alar, China; 5 Key Laboratory of Genetic Improvement and Efficient Production of Special Crops in Arid Areas, Tarim University, Alar, China

**Keywords:** fragrant pear, fruit cell tissue structure, fruit quality improvement, water and fertilizer ratio, yield

## Abstract

**Introduction:**

Optimizing water and fertilizer management is critical for improving fruit yield and quality in extreme arid regions, yet the synergistic effects of irrigation and NPK fertilization on fragrant pear (*Pyrus sinkiangensis*) remain inadequately characterized. This study aimed to identify the optimal water‑fertilizer strategy for fragrant pear cultivation in China's extreme arid regions through orthogonal experimental design.

**Methods:**

An orthogonal field experiment was conducted using an L9(33×21) mixed-level orthogonal array design. The experiment evaluated two irrigation levels (2400 and 3600 m3·ha-1) and three levels each of nitrogen (N: 150, 300, and 450 kg·ha-1), phosphorus (P₂O₅: 112.5, 225, and 337.5 kg·ha-1), and potassium (K₂O: 55, 110, and 165 kg·ha-1), resulting in nine treatment combinations. Measurements included fruit yield, quality parameters (soluble sugar, titratable acid, soluble solids, stone cell content, and firmness), cell tissue structure (pulp cell area and cellular arrangement), and anatomical features (stratum corneum, epidermis, and subepidermal thickness). Multivariate analysis was performed using principal component analysis (PCA).

**Results:**

The yield of fragrant pear was highest under T4 treatment (N-P2O5-K2O: 450-337.5-165 kg·ha−1, irrigation volume 2400 m3·ha−1). T6 treatment (N-P2O5-K2O: 300-337.5-55 kg·ha−1, irrigation volume 2400 m3·ha−1) achieved the most consistent fruit shape development pattern, as evidenced by its strong correlation with the least square fitting curve (R2 = 0.97626), reflecting optimal growth synchronization. The soluble sugar content was significantly higher under treatment T4 than under other treatments (P < 0.05), while treatment T3 (N-P2O5-K2O: 150-225-165 kg·ha−1, irrigation volume 2400 m3·ha−1) had a relatively high titratable acid content (0.17%), soluble solids content (12.23%), stone cell content (1.16%), and fruit firmness (7.17 kgf). Histological analysis revealed significant differences in cellular morphology among treatments. T1 and T5 treatments showed the largest mean pulp cell section area (0.015 mm2), and microscopic examination revealed that T3 treatment had a more compact cellular arrangement and improved pulp texture characteristics. Anatomical analysis showed that T6 treatment had an optimal structural configuration, with the stratum corneum (9.5 µm), epidermis (45.7 µm), and subepidermal layer (50.4 µm) demonstrating a thickness ratio of 1:4.8:5.3. Based on multivariate analysis through principal component analysis, T3 treatment (N-P2O5-K2O: 150-225-165 kg·ha−1, irrigation volume 2400 m3·ha−1) demonstrated superior performance across multiple evaluation parameters, particularly cellular development attributes and integrated quality indices.

**Conclusion:**

T3 treatment (N-P2O5-K2O: 150-225-165 kg·ha−1, irrigation volume 2400 m3·ha−1) emerged as the optimal water‑fertilizer strategy for high‑quality fragrant pear production in China's extreme arid regions, balancing yield, fruit quality, and cellular structural integrity. These findings provide a practical cultivation guideline for arid‑zone pear orchards.

## 1 Introduction

Chinese fragrant pear (*Pyrus sinkiangensis* ‘Korla’), a geographically protected cultivar within the white pears of the Rosaceae family, represents a significant horticultural resource in northwest China. This cultivar, characterized by its distinctive aromatic profile and exceptional postharvest longevity, has emerged as a cornerstone species, supporting the regional specialty fruit industry (Wu et al., 2007). According to recent industry statistics, the fragrant pear cultivation area in China has significantly expanded, reaching 2.67×10^4^ hm^2^ in 2024. Production data indicate an annual output exceeding 1.3 million metric tons, which represents a remarkable 40% increase. These metrics demonstrate a consistent upward trajectory in the industry’s scale and production capacity (Zhao, 2024). The primary fragrant pear production region is located in the hyper-arid region of southern Xinjiang in China, where agricultural water resource utilization intensity has surpassed 70%, significantly exceeding the international ecological warning threshold of 40%. This severe water resource deficit is a critical constraint hindering the sustainable development of the regional fruit industry (Tillerd et al., 2023; Ying et al., 2023). Under these challenging conditions, the advancement of water-efficient irrigation technologies and the optimization of mineral nutrition management have become imperative for enhancing the quality and economic viability of the fragrant pear industry.

Mineral nutrition metabolism is the fundamental basis for fruit tree growth and quality. The essential elements nitrogen (N), phosphorus (P), and potassium (K) collectively regulate photosynthetic product allocation, enzymatic activity expression, and stress resistance signaling pathways, directly influencing fruit cell development and quality (Landi and Papadakis, 2021; Buld and Jarrett, 2015). In pear trees, N deficiency reduces the carbon assimilation capacity and significantly decreases the individual fruit weight (Singh et al., 2022; De et al., 2012; Jeet et al., 2016; Dai et al., 2019). P insufficiency delays phenological processes and inhibits sugar accumulation (Dawar et al., 2022), and K deficiency disrupts cellular osmotic regulation and compromises fruit storage performance (Cheng et al., 2022; Zheng et al., 1993). Consequently, establishing a precise water–fertilizer management system represents a crucial approach for resolving the yield–quality dilemma in fragrant pear production.

Water and fertilizer coupling technology is known to significantly improve the rhizosphere microenvironment and enhance resource utilization efficiency by synergistically regulating soil moisture and mineral nutrient availability (Hu et al., 2022; Gray et al., 2012). Integrated water-fertilizer drip irrigation systems have been shown to optimize rhizosphere nutrient availability, thereby improving root uptake efficiency and crop productivity (Kumar et al., 2025; Sun et al., 2015; Suman et al., 2014). In fruit tree cultivation, the benefits of this approach have been widely demonstrated. For instance, precision drip irrigation enhances vegetative growth and yield in apple cultivation (Mankotia and Sharma, 2024), while integrated water-nitrogen management optimizes growth and fruit production in ‘Meideng’ apples while minimizing soil nitrogen residues (Ro et al., 2014). Furthermore, the interaction between water and nitrogen fertilizer exerts a statistically significant influence on multiple growth and physiological parameters of apple plants, including biomass accumulation, chlorophyll content, single-fruit weight, and total yield; using an integrated multi-criteria decision-making framework combining AHP, CRITIC, and TOPSIS, the optimal water–nitrogen regime was identified as 34.56 g N per plant coupled with irrigation at 82.3% of field capacity (Zhou HM et al., 2024). In pear orchard studies, drip irrigation coupled with water and fertilizer regulation reduced soil base ion content by 8.21% while increasing fruit yield and quality in Zaoyu pear (Lu et al., 2025), and optimizing irrigation to 515 mm with N application at 200 kg·ha^−1^ mitigated salt accumulation in the active root zone, leading to a 19.6% yield increase and a 1.6% increase in soluble solids content in high-density pear orchards (Liu et al., 2025). Beyond whole-plant responses, studies have also elucidated the cellular mechanisms underlying these effects. In sugarcane, coordinated water and fertilizer management increased sugar accumulation efficiency by 23.6% and the partial factor productivity of N fertilizer to 58.7 kg·kg^−1^ (Wu et al., 2022). More directly relevant to fruit texture and structural integrity, the combined application of compound fertilizer, organic fertilizer, and biological fertilizer effectively delays postharvest fruit softening by preserving fruit firmness, retarding the degradation of key cell wall polysaccharides (i.e., pectin and cellulose), and suppressing the enzymatic activities of polygalacturonase (PG), pectin methylesterase (PME), and cellulase (Cx), thereby sustaining fruit cell wall integrity through modulation of cell wall metabolism (Zhang Q et al., 2022). Notably, water and fertilizer regulation has been demonstrated to significantly enhance the yield and fruit quality of fragrant pear trees in the arid regions of Xinjiang (Li et al., 2024).

Previous studies on water and fertilizer management in pear and other woody fruit trees have primarily focused on whole-tree responses (yield and bulk quality parameters), largely overlooking the dynamic development of fruit tissue structure as a mediator between agronomic inputs and final quality. Furthermore, research on fragrant pear has been predominantly conducted in relatively fertile oasis agroecosystems, while the extreme arid regions of China (e.g., Xinjiang)—characterized by severe water scarcity and low soil organic matter—remain critically understudied. In contrast, the present study provides three new contributions: (1) it establishes a direct structural–quality linkage by investigating how water–N–P–K coupling affects fruit tissue structure throughout the main growth period; (2) it is specifically conducted in an extreme arid region (Xinjiang) under drip irrigation; and (3) it proposes a low-input, scientifically optimized water–fertilizer regime tailored to region-specific constraints. Unlike endpoint-only assessments, this research reveals that water–fertilizer regulation improves fruit quality by modulating tissue-level architecture (e.g., cell morphology and packing) during fruit development.

## 2 Materials and methods

### 2.1 Summary of the experimental sites

The experiment was conducted in 2022–2023 in the 29th Group of Tiemenguan City, Xinjiang Province, China (E85°31’, N41°47’). The soil used in this study was taken from 0–60 cm soil profile and classified as sandy loam type. The sandy loam had pH of 7.60, electric conductivity of 882 μs·cm^−1^, organic matter of 13 g·kg^−1^, salinity of 4.88g·kg^−1^, nitrate nitrogen of 19.04 mg·kg^−1^, ammonium nitrogen of 8.90 mg·kg^−1^, rapidly available phosphorus of 11.7 mg·kg^−1^, rapidly available potassium of 259.5mg·kg^−1^. The basic physical and chemical properties are shown in **Table 1**.

**Table 1:** Fertility of the test site.

Indicators	Soil depth			
Index	0–20	20–40	40–60	60–80
pH	7.56	7.58	7.62	7.63
Electric conductivity (μs·cm−1)	785	669	870	1205
Organic matter (g·kg−1)	18.45	18.23	7.65	7.65
Salinity (g·kg−1)	4.83	4.25	4.20	6.25
Nitrate nitrogen (mg·kg−1)	16.80	19.64	24.33	15.38
Ammonium nitrogen (mg·kg−1)	9.91	9.77	8.24	7.67
Rapidly available phosphorus (mg·kg−1)	25.10	11.13	6.61	3.96
Rapidly available potassium (mg·kg−1)	261	257	275	245

### 2.2 Test materials and design

**Figure 1:**
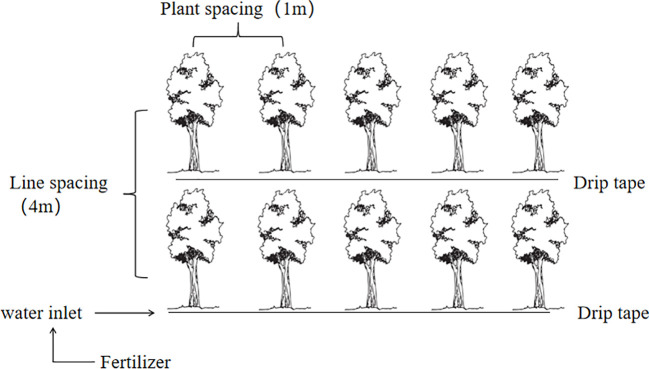
Fragrant pear planting pattern diagram.

As shown in **Figure 1**, eight-year-old grafted fragrant pear trees (*Pyrus sinkiangensis* ‘Korla’) were used as the test material, and the rootstock is *Pyrus betulifolia*. The spacing between plants and rows is 1×4m. The flowering, fruiting, and overall growth and development of a tree depend primarily on its nutrient reserves accumulated in the previous year. To ensure consistency in the experiment, we selected fragrant pear trees with similar tree height and crown width as our study subjects.

The experiment employed a mixed-level orthogonal design, specifically an L9(3^3^×2^1^) orthogonal array, involving four experimental factors: irrigation volume, nitrogen application rate, phosphorus application rate, and potassium application rate. Among them, nitrogen (N), phosphorus (P), and potassium (K) were three-level factors, while irrigation volume (W) was a two-level factor. The levels of each factor were determined based on previous experimental studies and relevant literature (Li et al., 2024; Chai et al., 2013; Liang and Zhou, 2008). Irrigation volume was set at two levels: 2400 m^3^·ha^-1^ (W1) and 3600 m^3^·ha^-1^ (W2). Nitrogen application rate was set at three levels: 150 kg·ha^-1^ (N1), 300 kg·ha^-1^ (N2), and 450 kg·ha^-1^ (N3). Phosphorus application rate was also set at three levels: 112.5 kg·ha^-1^ (P1), 225 kg·ha^-1^ (P2), and 337.5 kg·ha^-1^ (P3). Similarly, potassium application rate was set at three levels: 55 kg·ha^-1^ (K1), 110 kg·ha^-1^ (K2), and 165 kg·ha^-1^ (K3). The combination of experimental treatments is shown in **Table 2**. The use of a mixed-level orthogonal design allows for the evaluation of different factor levels on fruit tissue structure and quality of fragrant pears while reducing the number of experimental runs. Irrigation volume was set at only two levels based on local irrigation practices and preliminary experimental results, aiming to compare deficit and full irrigation effects under the premise of meeting basic tree water requirements, while avoiding an excessive increase in the experimental scale caused by too many levels.

**Table 2:** L9(3^3^×2^1^) orthogonal test treatment combinations.

Test treatment	Irrigation volume	Nitrogen fertilizer	Phosphate fertilizer	Potassium fertilizer
T1	W1	N1	P1	K1
T2	W1	N2	P2	K2
T3	W1	N1	P2	K3
T4	W1	N3	P3	K3
T5	W1	N3	P1	K2
T6	W1	N2	P3	K1
T7	W2	N2	P1	K3
T8	W2	N1	P3	K2
T9	W2	N3	P2	K1

In this experiment, four fertilizer applications were performed via drip irrigation, with drip lines positioned 40 cm from both sides of the pear trees. Fertilization was carried out at four phenological stages: flowering and leaf expansion (April to early May), fruit development (mid-May to early June), fruit growth (mid-June to late July), and maturity (early August). The proportions of nitrogen applied at these stages were 30%, 25%, 25%, and 20%, respectively; phosphorus was applied at 30%, 30%, 20%, and 20%; and potassium at 20%, 20%, 30%, and 30%. The two irrigation levels, W1 (2400 m^3^·ha^-1^) and W2 (3600 m^3^·ha^-1^), were each applied six times per year, with respective single irrigation volumes of 400 m^3^·ha^-1^ and 600 m^3^·ha^-1^. These six irrigation applications were evenly distributed across the four phenological stages: one during flowering and leaf expansion, two during fruit development, two during fruit growth, and one at maturity.

The field experiment was arranged according to an L9(3^3^×2^1^) mixed-level orthogonal design, consisting of nine treatments. Each treatment was replicated three times in a randomized complete block design (RCBD), resulting in a total of 27 experimental plots. For each treatment, within a single replicate, the same irrigation level was applied to two adjacent rows of fragrant pear trees. Consequently, across the three replicates, each treatment covered a total of six rows of trees.

Each experimental plot contained ten fragrant pear trees, of which the central five trees were designated as sampling units (observational units), and the surrounding five trees served as border rows to minimize edge effects. The experimental unit for statistical analysis was defined as the plot mean calculated from the five sampled trees. All statistical comparisons were performed using these plot means as independent data points (n = 3 replicates × 2 years = 6 data points per treatment).

### 2.3 Index determination method

#### 2.3.1 Determination of the yield of fragrant pear trees

In each experimental unit, three non-adjacent trees with comparable growth status were randomly selected for fruit sampling. All fruits from the selected trees were harvested and individually weighed. The yield per hectare was then calculated based on the collected data.

#### 2.3.2 Determination of the fruit shape index

In each experimental unit, four non-adjacent trees were randomly selected. From each selected tree, 10 fruits were randomly sampled from the eastern, southern, western, and northern directions to serve as fixed measurement samples. Measurements were then conducted at 10-day intervals.

The fruit shape index was calculated as follows:


Fruit shape index = fruit longitudinal diameter/fruit transverse diameter

#### 2.3.3 Collection and determination of fruit quality indexes

During fruit maturity, 30 fresh fruits were randomly selected from each experimental unit. The fruit hardness (fruit hardness tester), fruit stone cell content (freezing method), fruit soluble solids content (handheld saccharometer), soluble sugar content (concentrated sulfuric acid-anthrone colorimetric method), and titratable acidity (ethanol extraction–lye titration method) were determined.

#### 2.3.4 Determination of pulp cell development

**Figure 2:**
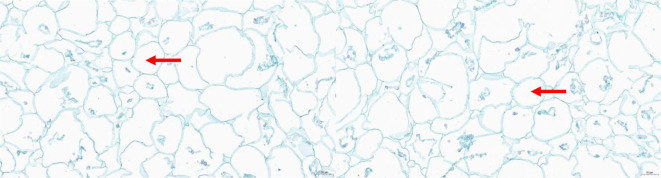
Selected field of view of pulp cells. The red arrows indicate the measured pulp cell area.


In each experimental unit, three non-adjacent fruit trees were selected. At the fruit development, fruit expansion, and fruit ripening stages, 20 fruits were randomly selected from each tree and stored in a formaldehyde–alcohol–acetic acid (FAA) fixative for testing. A PANNORAMIC slide scanner was used for section imaging. After imaging, Image-Pro Plus 6.0 analysis software was used. A uniform standard unit of millimeters was adopted. A 200× field of view was selected to determine the number of flesh cells in 3 fields of view for each section (**Figure 2**), and the area of the field of view was measured. The average area of the flesh cells was calculated as follows:

Average area of flesh cells = field of view area/number of flesh cells

#### 2.3.5 Determination of pericarp cell development

**Figure 3:**
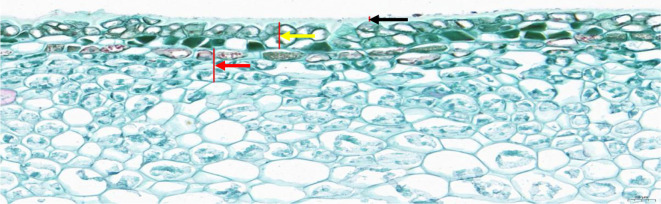
Selected field view of pericarp cells. The black arrow show the thickness of the stratum corneum; the yellow arrow show the skin thickness; and the red arrow show the subepidermal thickness.

As shown in **Figure 3**, for imaging during flesh cell development, Image-Pro Plus 6.0 analysis software was used to select a 400× field of view, and the thickness was measured in 5 cuticle layers, 5 epidermal cells, and 5 subepidermal cells in each section.

### 2.4 Data processing and analysis

The data processing is based on the average values derived from the 2022-2023 dataset. Preliminary data processing was performed using Microsoft Excel 2015, and SPSS 26.0 (IBM Corporation, Armonk, NY, USA) was employed for analysis of variance (ANOVA) with significance levels set at P < 0.05. Data visualization, least square curve fitting, and principal component analysis (PCA) were conducted using OriginPro 2022 (OriginLab Corporation, Northampton, MA, USA).

## 3 Results

### 3.1 Yield in fragrant pear through integrated water–fertilizer management

**Figure 4:**
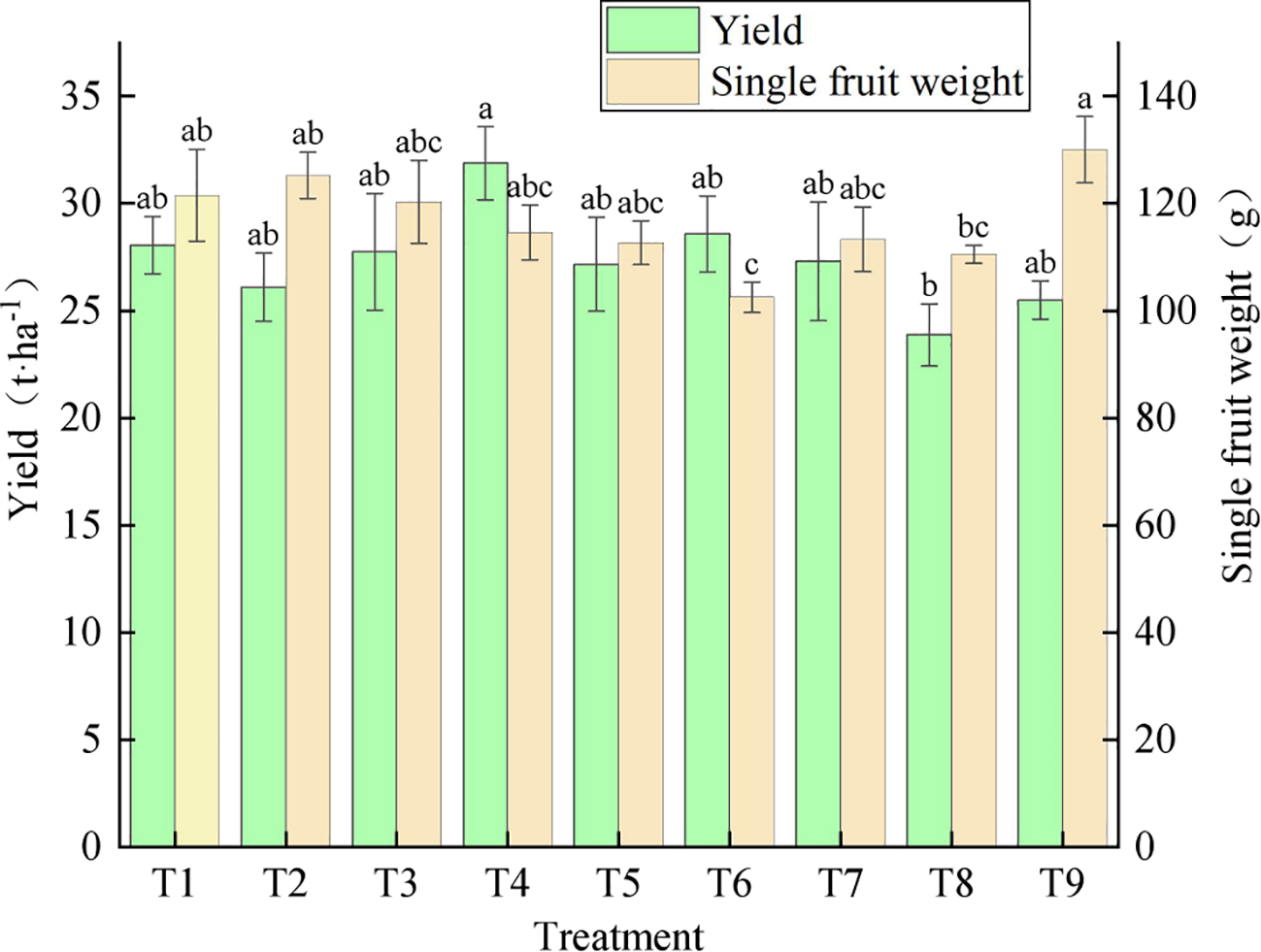
Yield under integrated water–fertilizer management conditions. The absence of common lowercase letters a, b, c and d indicate significant differences between treatments (*P* < 0.05).

The increase in fertilizer application significantly increased fragrant pear yield (**Figure 4**). The highest yield of Korla fragrant pears was observed under the T4 treatment, reaching 31.88 t·ha^-1^, while the lowest yield occurred under the T8 treatment, at only 23.87 t·ha^-1^, which was significantly lower than that under T4. In terms of single fruit weight, the T9 treatment resulted in the highest value of 130.08 g, significantly exceeding those recorded under the T6 and T8 treatments.

**Figure 5:**
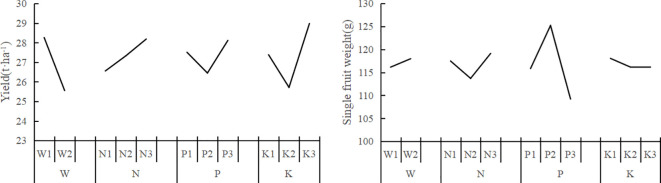
Yield, single fruit weight and single factor trends of fragrant pear.

As shown in **Figure 5**, range analysis revealed that for yield, the factor priority order was K>W>P>N, with the optimal level combination identified as W1N3P3K3; for single fruit weight, the factor priority order was P>N>K>W, leading to the optimal combination of W2N3P2K1.

### 3.2 Dynamic regulation of fruit morphogenesis in fragrant pear through integrated water–fertilizer management

**Figure 6:**
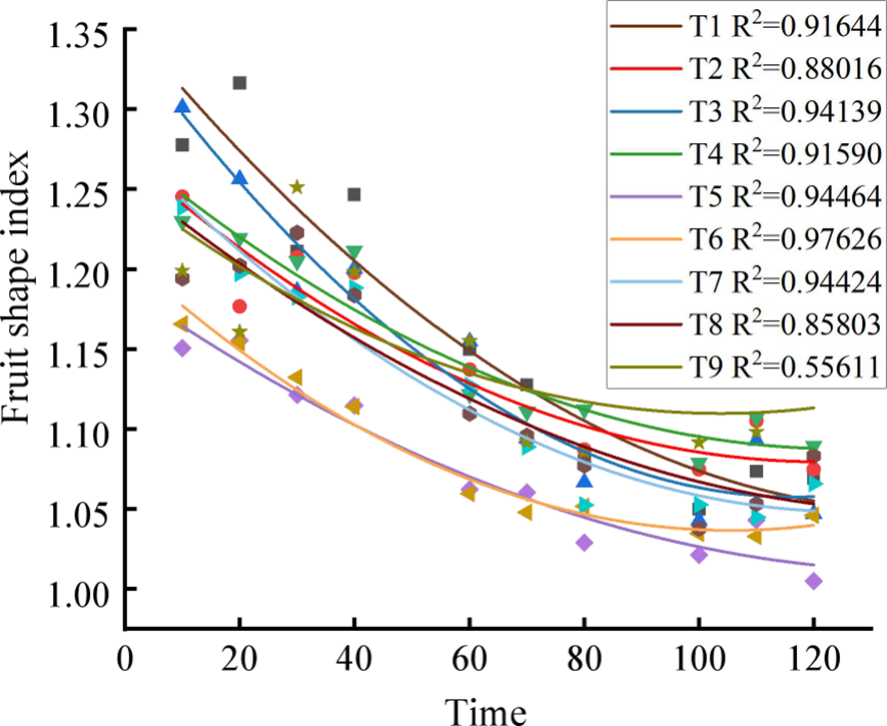
Dynamic fitting curve of the pear fruit shape index under integrated water–fertilizer management conditions.

The fruit shape index serves as a crucial quality parameter in commercial fruit evaluation. The growth dynamic equilibrium was quantitatively assessed by establishing a regression model with the fruit growth time as the independent variable and the fruit shape index as the dependent variable. As shown in **Figure 6**, the coefficient of determination (R^2^) of the regression fitting curves showed the following order for the integrated water and fertilizer treatments: T6 (0.97626) > T5 (0.94464) > T7 (0.94424) > T3 (0.94139) > T1 (0.91644) > T4 (0.9159) > T2 (0.88016) > T8 (0.85803) > T9 (0.55611). In terms of goodness of fit, the growth trajectory of the fruit shape exhibited the closest alignment with the fitted growth curve under T6 treatment, with an R^2^ value approaching 1. Consequently, a moderate N application (300 kg·ha^−1^) combined with a low irrigation volume (2400 m^3^·ha^−1^) is recommended to effectively synchronize the growth of the vertical and horizontal diameters of fragrant pear.

### 3.3 Water–fertilizer coupling differentially regulates fruit quality indices

**Figure 7:**
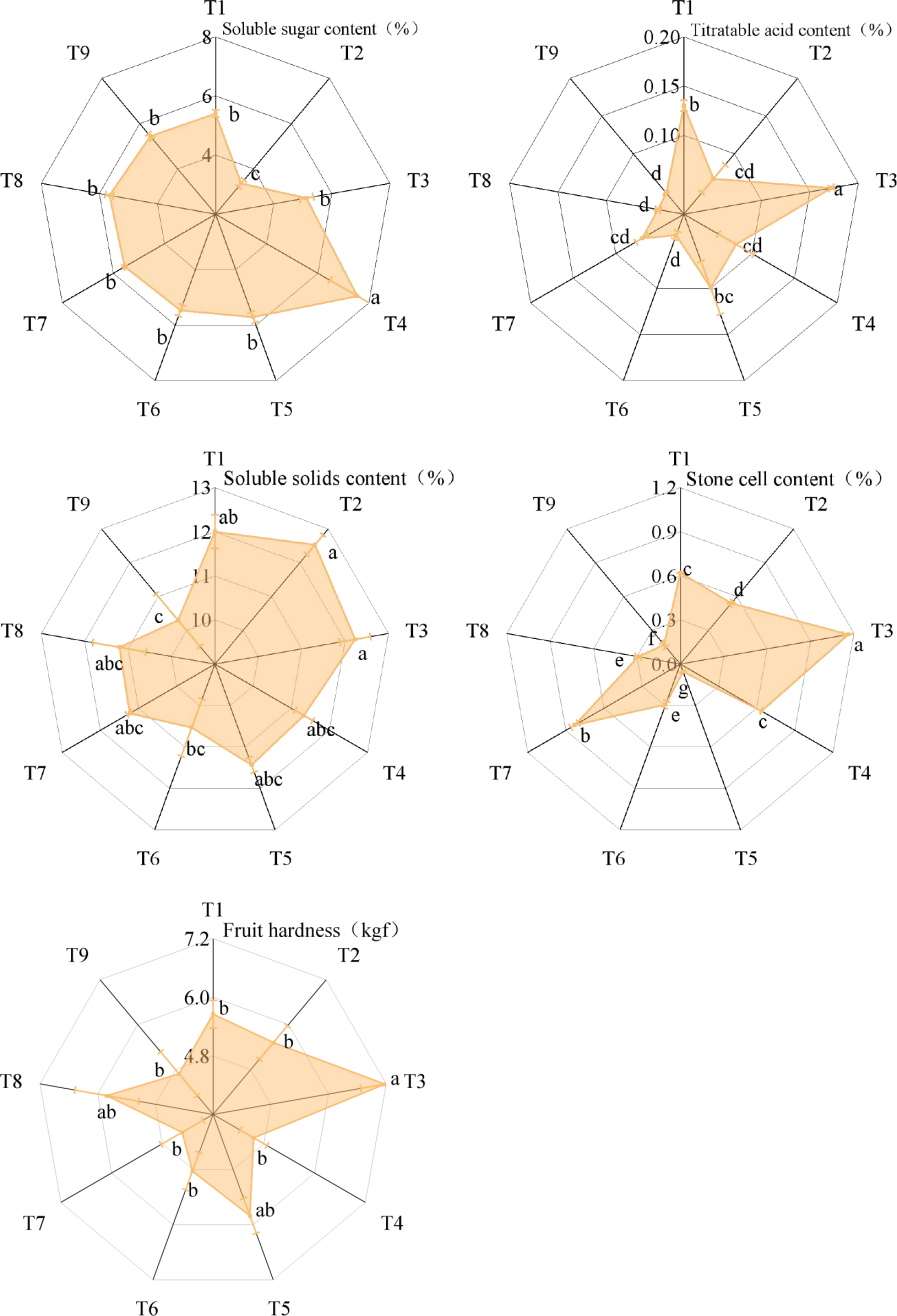
Effects of water–fertilizer interactions on the quality attributes of fragrant pear fruit.

The fruit quality index serves as a critical determinant in assessing the commercial value and application potential of fruit. As shown in **Figure 7**, when the application rates of nitrogen, phosphorus, and potassium fertilizers are all at their highest levels concurrently, the soluble sugar content in the fruit increases significantly. Conversely, when these fertilizer application rates are all at medium levels simultaneously, antagonistic interactions among nutrients may occur, leading to a marked reduction in the soluble sugar content of the fruit. T4 treatment had a significantly higher soluble sugar content than the other treatments, with a remarkable 125% increase over T2 treatment. The titratable acid content, which influences fruit taste, plays a pivotal role in determining fruit sweetness. Increasing irrigation and nitrogen application while reducing potassium supply can effectively decrease titratable acid content in fruits. Of the treatments, T3 had the highest titratable acid content. The soluble solids content, representing the total carbohydrate concentration in fruit, was highest under T2 treatment (12.53%), followed by T3 treatment (12.23%). In terms of stone cell content, T3 treatment significantly outperformed all of the other treatments. Fruit hardness, a direct indicator of ripening degree, is inversely correlated with ripeness and the water content. The strategic increase in the application rate of nitrogen and phosphorus fertilizers, coupled with a controlled reduction in irrigation levels, can significantly enhance the post-harvest storage duration of fruits. T3 treatment exhibited the highest fruit hardness (7.17 kgf), significantly surpassing all of the treatments except T5 and T8. Compared to T7, T3 demonstrated a substantial 65.6% increase in fruit hardness. These findings collectively highlight the distinct effects of different treatments on fruit quality parameters.

**Figure 8:**
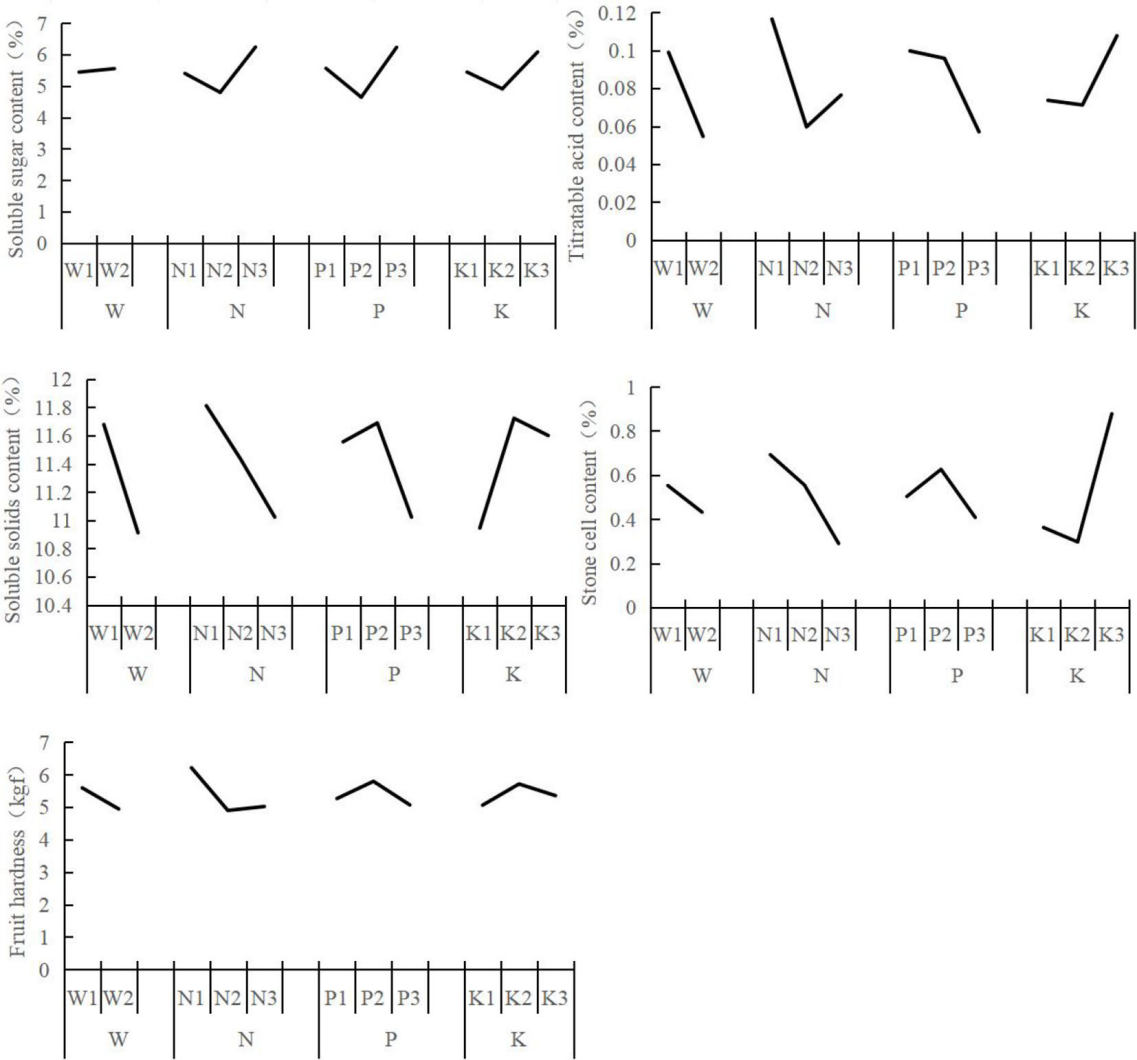
Soluble sugar content, titratable acid content, soluble solids content, stone cell content, fruit hardness and single factor trends of fragrant pear.

As shown in **Figure 8**, range analysis revealed that for soluble sugar content, the factor priority order was P>N>K>W, with the optimal level combination identified as W2N3P3K3; for titratable acid content, the factor priority order was N>W>P>K, leading to the optimal combination of W2N2P3K2; for soluble solids content, the factor priority order was N>W>K>P, leading to the optimal combination of W1N1P2K2; for stone cell content, the factor priority order was K>N>P>W, leading to the optimal combination of W2N3P3K2; for fruit hardness, the factor priority order was N>P>K>W, leading to the optimal combination of W1N1P2K2.

### 3.4 Temporal and spatial impacts of water and fertilization coupling on fruit cell development

#### 3.4.1 Morphogenetic patterns and cellular architecture of fruit pulp tissue

**Figure 9:**
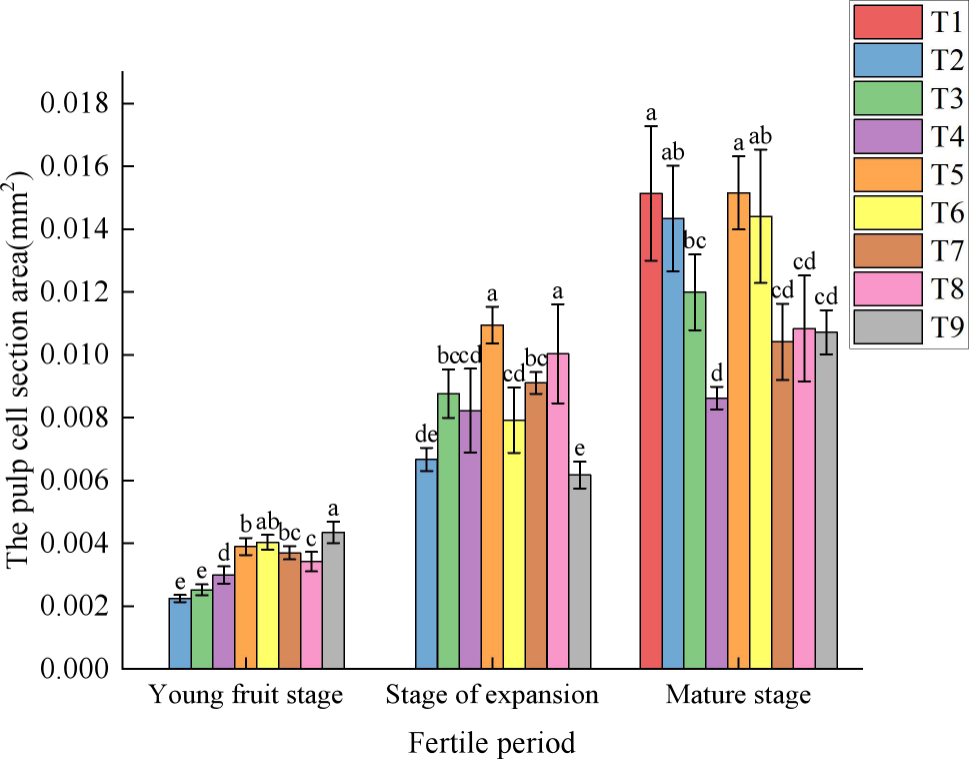
Effects of water and fertilizer coupling on the average pulp cell area in fragrant pear.

As shown in **Figure 9**, the pulp cell section area of the pear fruit exhibited a gradual increasing trend throughout the growth period. During the young fruit development stage, T6 and T9 treatments showed a significantly larger pulp cell section area than the other treatments. In the fruit expansion stage, T5 and T8 treatments outperformed all of the other treatments except T1, while the pulp cell section area of the remaining treatments (excluding T1, T5, and T8) developed at a slower rate. At the maturity stage, T1 and T5 treatments had a significantly larger pulp cell section area (0.015 mm^2^) than the other treatments, except for T2 and T6. These findings suggest that increasing the fertilizer application rate or irrigation volume does not necessarily enhance fruit quality by promoting pulp development. Instead, an appropriate water and fertilizer balance is crucial for regulating pulp cell development in fragrant pear, thereby optimizing fruit quality to some extent.

**Figure 10:**
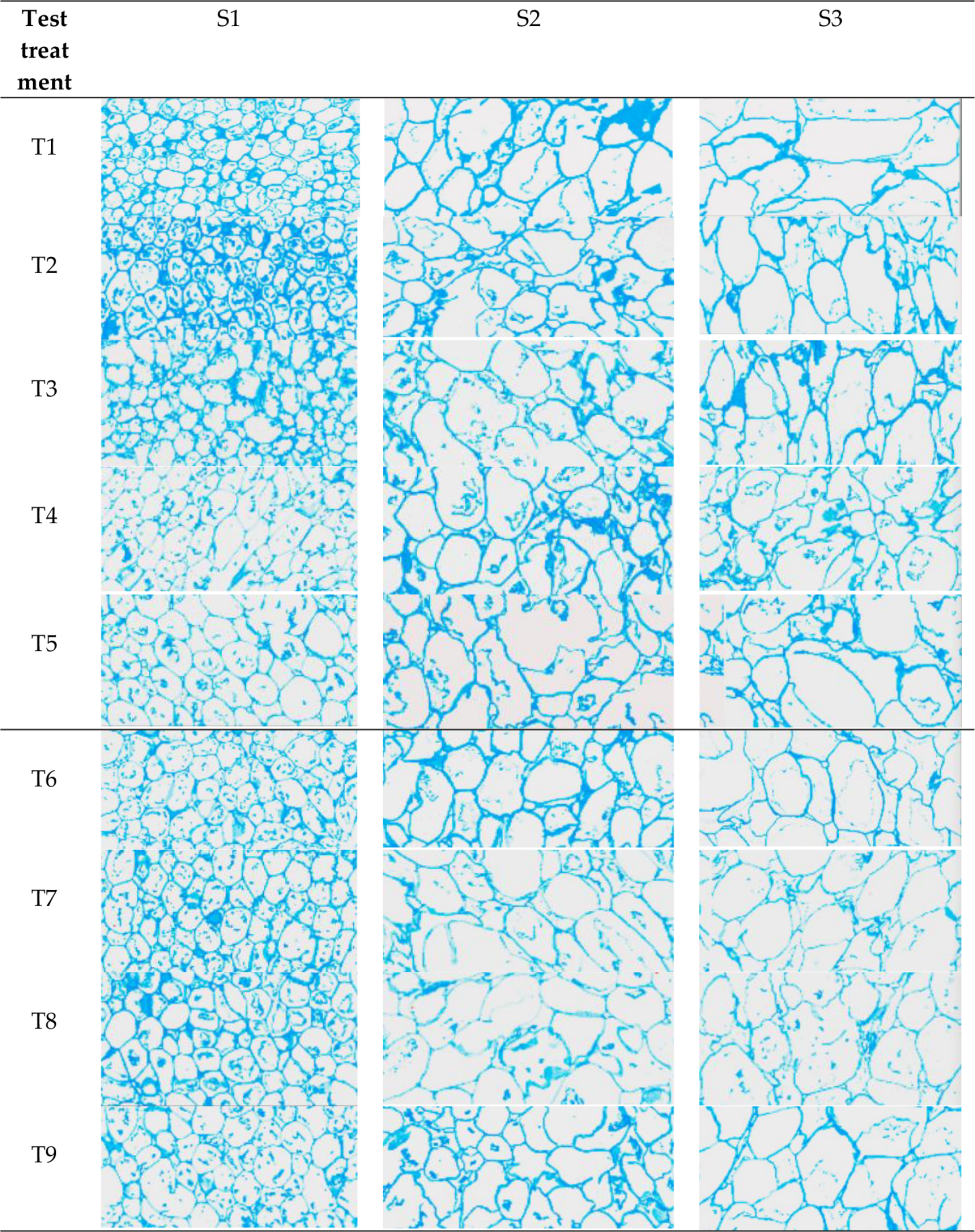
Microstructure of fruit pulp cells during fragrant pear development under water and fertilizer coupling. S1 is the young fruit development stage; S2 is the fruit expansion stage; and S3 is the fruit ripening stage.

As shown in **Figure 10**, the growth trend of fragrant pear pulp cells became increasingly evident as the growth period progressed. During the young fruit development stage, the number of pulp cells in treatments T1, T2, T3, T8, and T9 was significantly higher than that in the other treatments. In addition, the pulp cells in treatments T1, T2, and T3 were more densely arranged, although they were smaller in size than those in the other treatments. In the fruit expansion stage, the pulp cell size increased most significantly under T1 and T5 treatments, while the pulp cells exhibited a more compact arrangement under T2 and T4 treatments. At the mature stage, the pulp cells were more closely arranged under T3 and T4 treatments; however, the cell size under T4 treatment did not show a significant increase compared to the previous stage. As the irrigation amount increased, the gaps between pulp cells tended to widen in fragrant pear. Conversely, under high fertilizer application rates, pulp cell growth was not as pronounced. These observations highlight the nuanced effects of irrigation and fertilization on pulp cell development and arrangement in fragrant pear.

#### 3.4.2 Characteristics of pericarp stratification development

As shown in **Table 3**, peel development in fragrant pear exhibited a gradual upward trend with increasing fertilizer application. Throughout the pear tree growth period, peel development followed a pattern of initial increase, followed by a decrease. During the young fruit development stage, the stratum corneum thickness was significantly greater under T6 treatment than under the other treatments, while the epidermal and subepidermal thicknesses were significantly higher under T5 treatment than under the other treatments. In the fruit expansion stage, T5 treatment had a significantly greater stratum corneum thickness than the other treatments. At the same stage, T7 treatment showed a significantly higher epidermal thickness, and T8 treatment exhibited a significantly greater subepidermal thickness. At the ripening stage, T6 treatment had a significantly higher stratum corneum thickness, epidermal thickness, and subepidermal thickness than the other treatments, with the least change observed compared to the fruit expansion stage. Overall, the thickness ratio of the stratum corneum (9.5 µm), epidermis (45.7 µm), and subepidermis (50.4 µm) under T6 treatment was approximately 1:4.8:5.3. These findings indicate that water and fertilizer regulation promote, to some extent, the development of peel cells in fragrant pear, thereby influencing fruit texture and quality.

**Table 3:** Effects of water and fertilizer management on peel development in fragrant pear.

Fertile period	Test treatment	Stratum corneum thickness (µm)	Epidermal thickness (µm)	Subepidermal thickness (µm)
Young fruit development period	T1	8.3 ± 0.3b	29.3 ± 1.7c	52.1 ± 3.5a
	T2	8.6 ± 1.6ab	35.1 ± 1.9b	43.6 ± 3.0bc
	T3	4.9 ± 0.9d	24.9 ± 3.3de	30.2 ± 3.6e
	T4	7.5 ± 0.4bc	23.5 ± 3.3de	29.1 ± 3.8e
	T5	8.7 ± 1.1ab	45.1 ± 2.2a	52.3 ± 3.7a
	T6	9.6 ± 0.8a	35.4 ± 4.4b	45.9 ± 2.0b
	T7	7.0 ± 0.9c	21.0 ± 3.4e	37.1 ± 3.7d
	T8	5.3 ± 0.6d	25.8 ± 0.8cd	31.3 ± 2.4e
	T9	5.9 ± 0.5d	27.0 ± 4.3cd	38.2 ± 9.1d
Fruit expansion period	T1	6.9 ± 1.1c	33.9 ± 0.9de	29.4 ± 4.1e
	T2	8.5 ± 1.4b	55.4 ± 2.9b	49.8 ± 6.5bc
	T3	5.3 ± 0.5de	36.5 ± 3.2d	27.2 ± 3.4e
	T4	5.1 ± 0.3de	26.1 ± 3.7f	32.4 ± 5.9e
	T5	11.8 ± 0.7a	50.0 ± 6.4c	25.9 ± 3.9e
	T6	8.5 ± 1.7b	35.2 ± 1.7d	41.0 ± 5.3d
	T7	6.1 ± 0.4cd	60.4 ± 2.7a	53.9 ± 8.6b
	T8	4.2 ± 0.8e	29.7 ± 2.2ef	68.1 ± 3.2a
	T9	6.2 ± 0.8cd	35.8 ± 3.9d	43.9 ± 8.6cd
Fruit Ripening period	T1	6.9 ± 0.8cde	27.6 ± 4.3c	31.9 ± 5.0cd
	T2	7.8 ± 0.9bc	33.5 ± 4.1b	25.4 ± 2.0de
	T3	7.2 ± 1.3bcd	46.6 ± 5.9a	30.8 ± 4.1cd
	T4	8.5 ± 1.3ab	24.6 ± 1.4c	32.3 ± 4.2c
	T5	6.5 ± 0.6cde	22.4 ± 4.1c	20.5 ± 2.5e
	T6	9.5 ± 1.2a	45.7 ± 3.9a	50.4 ± 4.5a
	T7	5.7 ± 0.7e	25.8 ± 2.9c	41.3 ± 8.9b
	T8	6.6 ± 1.8cde	34.1 ± 0.9b	28.0 ± 5.1cd
	T9	6.2 ± 0.4de	27.5 ± 2.8c	33.9 ± 1.8c

Lowercase letters a, b, c, and d indicate significant differences between treatments (*P* < 0.05).

### 3.5 Interplay between cellular development and fruit quality parameters

**Figure 11:**
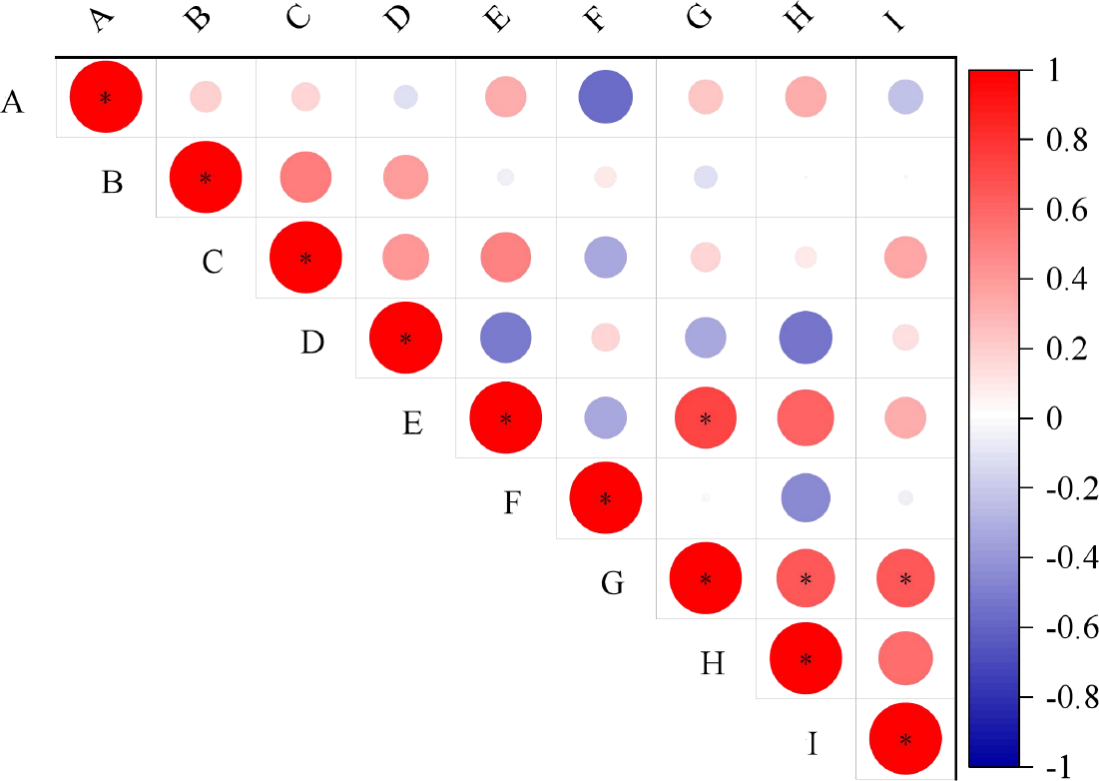
Correlation analysis between cell development and fruit quality in fragrant pear. (A) is the average pulp cell area; (B) is the stratum corneum thickness; (C) is the epidermal thickness; (D) is the subepidermal thickness; (E) is the fruit hardness; (F) is the soluble sugar content; (G) is the titratable acid content; (H) is the soluble solids content; and (I) is the stone cell content. *p<0.05.

The cellular development of fragrant pear significantly influenced fruit quality, as shown in **Figure 11**. Our analysis revealed an inverse relationship between the average pulp cell area and the soluble sugar content, indicating that cellular expansion dilutes sugar accumulation. In addition, we observed a positive correlation between epidermal thickness and fruit hardness, suggesting that cuticle layer deposition enhances the fruit’s mechanical strength. Subepidermal thickness was negatively correlated with the soluble solids content, indicating its inhibitory effect on organic substance synthesis pathways. The stone cell content showed a positive correlation with the skin thickness but a negative correlation with the average flesh cell size. Collectively, these findings suggest that optimal fragrant pear quality, characterized by superior taste, is associated with thinner skin, larger flesh cells, and a reduced stone cell content.

### 3.6 Effects of the water and fertilizer coupling system based on principal component analysis

Principal component analysis was performed on the average flesh cross-sectional area, peel thickness, soluble sugar content, titratable acid content, soluble solids content, stone cell content, and fruit firmness of Chinese fragrant pear. As shown in **Table 4**, based on the criterion of an eigenvalue greater than 1, a total of three principal components were extracted, with a cumulative variance contribution rate of 82.668%, which can represent most of the information regarding the fruit tissue structure and quality of fragrant pear. According to the eigenvectors presented in **Table 4**, three score expressions were obtained by multiplying each eigenvector by the corresponding standardized indicator value and summing the products.

**Table 4:** Eigenvectors, eigenvalues, and contribution rates of the three principal components influencing fruit tissue structure and quality of Chinese fragrant pear.

Factor	Principal component		
	F1	F2	F3
X1	0.252	-0.610	0.072
X2	-0.021	0.069	0.898
X3	0.481	-0.020	-0.040
X4	-0.277	0.534	-0.246
X5	0.489	0.269	-0.122
X6	0.512	-0.005	-0.176
X7	0.357	0.514	0.282
Eigenvalue	3.017	1.592	1.178
Variance contribution rate/%	43.103	22.739	16.826
Cumulative variance contribution rate/%	43.103	65.842	82.668

F1 = 0.252X1-0.021X2 + 0.481X3-0.277X4 + 0.489X5 + 0.512X6 + 0.357X7;

F2=-0.61X1 + 0.069X2-0.02X3 + 0.534X4 + 0.269X5-0.005X6 + 0.514X7;

F3 = 0.072X1 + 0.898X2-0.04X3-0.246X4-0.122X5-0.176X6 + 0.282X7.

The variance contribution rates of the principal components were adopted as weights to establish a comprehensive evaluation equation for the tissue structure and fruit quality of Chinese fragrant pear: F = 0.43103F1 + 0.22739F2 + 0.16826F3. The comprehensive score for each treatment was subsequently calculated using this equation. As shown in **Table 5**, the comprehensive evaluation of fruit tissue structure and fruit quality of fragrant pears ranked in the following order: T3 > T1 > T2 > T7 > T4 > T8 > T6 > T5 > T9. This indicates that the T3 treatment (N-P_2_O_5_-K_2_O: 150-225-165 kg·ha^−1^, irrigation volume 2400 m^3^·ha^−1^) is more favorable for fruit cell development and the formation of sugar and acid quality in fragrant pear.

**Table 5:** Comprehensive analysis of various indexes for fragrant pear under water and fertilizer coupling treatments.

Test treatment	Comprehensive evaluation				Ranking
	F1	F2	F3	F	
T1	1.492	−0.315	−0.409	0.503	2
T2	1.451	−1.660	0.051	0.257	3
T3	3.241	1.423	0.811	1.857	1
T4	−1.315	2.109	−0.800	−0.222	5
T5	0.209	−1.261	−1.707	−0.484	8
T6	−1.430	−0.929	2.182	−0.461	7
T7	−0.758	0.912	0.368	−0.057	4
T8	−0.836	0.068	−0.336	−0.401	6
T9	−2.054	−0.348	−0.161	−0.991	9

## 4 Discussion

### 4.1 Regulatory mechanisms of water–fertilizer coupling on fragrant pear fruit growth and development

Scientific and rational water–fertilizer management is a key strategy for enhancing fruit tree growth (Li et al., 2021). This study evaluated the effects of different water–fertilizer combinations on eight-year-old grafted fragrant pear trees. Under an irrigation volume of 2400 m^3^·ha^-1^ (W1), an optimized fertilizer ratio (T3: N-P_2_O_5_-K_2_O of 150-225-165 kg·ha^-1^) maintained yield while significantly improving fruit quality.

Previous studies have confirmed the regulatory role of optimized water–fertilizer coupling in fruit tree growth (Liu et al., 2020; Wang JX et al., 2004; Hou et al., 2016), consistent with our findings. However, compared with other irrigation districts in southern Xinjiang, the water and fertilizer rates recommended in this study are considerably lower. Wang et al. (2023) recommended irrigation of 6700–7200 m^3^·ha^-1^ and nitrogen of 300–375 kg·ha^-1^ in the Tarim River Basin, approximately 2.8–3 times and 2–2.5 times higher than those in this study, respectively. Zhang et al. (2022) recommended irrigation of 5400–6000 m^3^·ha^-1^ in the Aksu region, also significantly higher. Li (2024) proposed a lower irrigation threshold of 4800 m^3^·ha^-1^ for fragrant pear in the Hotan region, below which yield and fruit weight declined markedly. In contrast, the present study achieved acceptable yield and superior quality with only 2400 m^3^·ha^-1^.

These differences can be attributed to regional heterogeneity: (1) a relatively shallow groundwater table in the study area (upper Kongqi River, Bazhou) providing supplemental water; (2) higher baseline soil nitrate−nitrogen (32.4 mg·kg^−1^ in the 0–60 cm layer) compared with 15–25 mg·kg^−1^ in the Tarim Basin and Aksu region, reducing exogenous nitrogen demand; and (3) improved nutrient use efficiency through precise drip fertigation (Han et al., 2013; Levinson and Adato, 2015).

Furthermore, potassium fertilization significantly promoted fruit development, with high potassium rates outperforming moderate or low rates. This challenges the conventional assumption that potassium−rich Xinjiang soils do not require K supplementation, likely due to synergistic N−P−K interactions. Similar results have been reported for walnut cultivation in southern Xinjiang (Hu et al., 2021).

In summary, this study demonstrates that reducing irrigation and nitrogen inputs while appropriately increasing phosphorus and potassium fertilization can significantly enhance fragrant pear growth and yield. The proposed water−fertilizer regime features substantially lower application rates with higher efficiency, providing a valuable reference for sustainable fruit cultivation in arid regions.

### 4.2 Cytological mechanisms underlying water–fertilizer coupling effects on pear fruit quality development

Fruit quality is a critical determinant of commercial fruit value, with firmness, soluble solids content, and cellular structural features widely recognized as core indicators (Romero et al., 2024; Qu et al., 2024). Firmness is closely associated with pectin and cellulose contents in the fruit cell wall: increased pectin leads to softening, while cellulose maintains structural integrity (Paniagua et al., 2017; Zhang et al., 2015). Water–fertilizer coupling strategies can significantly improve fruit quality, with irrigation amount, fertilizer rate, and their interactions exerting differential effects (Porro et al., 2013).

Previous studies have shown that appropriate water-to-fertilizer ratios enhance photosynthesis and respiration, promoting high-quality fruit development (Sun et al., 2010; Zhang, 2021; Wang R. et al., 2024). The present study supports this view but further reveals that under reduced irrigation and nitrogen inputs, moderate increases in phosphorus and potassium can optimize cellular structure while maintaining firmness and soluble solids content (Thu et al., 2024; Pan et al., 2025; Shi et al., 2021). This differs from the common paradigm emphasizing “high water and high nitrogen for high yield.” For example, Porro et al. (2013) found that increasing nitrogen input was the main driver of yield in apples. In contrast, the present study demonstrates that under specific site conditions (shallow groundwater table, higher baseline soil fertility), adjusting fertilizer ratios rather than increasing total inputs can synergistically enhance fruit quality. A plausible biological explanation is that reduced nitrogen input alleviates excessive vegetative growth, redirecting photosynthetic assimilates toward reproductive organs.

The density of cellular arrangement is an intrinsic determinant of fruit taste and storage performance. In this study, certain treatments (e.g., T1 and T5) exhibited large, loosely arranged flesh cells, consistent with previously reported associations with poor sensory attributes and rapid softening (Zhang et al., 2024; Liu et al., 2024). In contrast, the T3 treatment achieved a denser cellular structure. Notably, Zhou Y et al. (2024) reported that increased flesh cell area is typically accompanied by higher juice content but dilution of soluble sugars. However, in the T3 treatment of this study, increased cell area did not compromise soluble solids content, suggesting that coordinated phosphorus and potassium supply may play a specific regulatory role in carbon partitioning (Cakmak, 2005; Römheld and Kirkby, 2010).

Balanced pericarp development also affects texture. In the T6 treatment, the thickness ratio of peel layers was approximately 1:4.8:5.3, with a total thickness of only 0.1056 mm, consistent with Giménez Bañón et al.’s (2024) observation that thinner peels improve taste. Dong et al. (2025) further noted that exogenous management practices can modulate peel cell division and expansion to influence textural properties, and the present study provides empirical support for this view.

The regulation of fruit quality by water–fertilizer coupling involves multiple physiological processes. First, balanced water and nutrient supply modulates cell wall metabolism, thereby affecting tissue firmness (Zhang et al., 2015; Brummell, 2006). Second, appropriate N-P-K ratios—particularly coordinated phosphorus and potassium supply under reduced nitrogen conditions—enhance carbon assimilation and optimize the partitioning of photosynthetic products between sugars and structural components (Porro et al., 2013; Cakmak, 2005). Furthermore, this study suggests that in regions with high baseline soil nitrogen and a shallow groundwater table, the marginal benefit of exogenous nitrogen diminishes, while the regulatory role of phosphorus and potassium becomes more prominent. This region−specific response may be explained by the fact that groundwater nitrates and residual soil nitrogen sufficiently meet crop nitrogen demand, allowing phosphorus and potassium to exert their quality-regulating functions without nitrogen-induced growth dilution (Ju et al., 2005; Liu et al., 2013).

In summary, under reduced water and nitrogen inputs, increasing phosphorus and potassium enhances pear fruit quality by optimizing cellular structure, balancing pericarp development, and regulating carbon partitioning. This lower-input, higher-efficiency strategy offers a sustainable alternative to conventional high-water, high-nitrogen regimes in arid regions.

## 5 Conclusions

Appropriate water and nitrogen regulation significantly improves the yield, fruit quality, pulp cell structure, and peel development of Chinese fragrant pear. Increased fertilization significantly enhanced yield, with the highest yield under T4 (31.88 t·ha^−1^) and the lowest under T8 (23.87 t·ha^−1^). The highest single fruit weight was recorded under T9 (130.08 g). The T6 treatment (N-P_2_O_5_-K_2_O: 300-337.5-55 kg·ha^−1^, irrigation volume 2400 m^3^·ha^−1^) exhibited the highest goodness-of-fit for fruit shape growth (R^2^ = 0.97626), most favorably coordinating vertical and horizontal diameter growth. Soluble sugar content was highest under T4, showing a 125% increase over T2. Titratable acid decreased with increasing water and nitrogen, with the highest value under T3. Soluble solids content was highest under T2 (12.53%). Stone cell content and fruit hardness (7.17 kgf, 65.6% higher than T7) were highest under T3. At maturity, the largest pulp cell cross-sectional area was observed under T1 and T5 (0.015 mm^2^). Increased irrigation expanded intercellular spaces, whereas excessive fertilization suppressed pulp cell growth. At maturity, T6 exhibited significantly greater stratum corneum (9.5 µm), epidermal (45.7 µm), and subepidermal (50.4 µm) thicknesses than other treatments, with an approximate ratio of 1:4.8:5.3. Pulp cell area was negatively correlated with soluble sugar content, while epidermal thickness was positively correlated with fruit hardness. High-quality fragrant pear is characterized by thin peel, large pulp cells, and low stone cell content. The comprehensive ranking of treatments was T3 > T1 > T2 > T7 > T4 > T8 > T6 > T5 > T9. Therefore, the T3 treatment (N-P_2_O_5_-K_2_O: 150-225-165 kg·ha^−1^, combined with an irrigation volume of 2400 m^3^·ha^−1^) is most favorable for fruit cell development and the formation of sugar and acid quality in Chinese fragrant pear.

In summary, for pear cultivation in the arid region of Xinjiang, China, water and fertilizer regimes should be flexibly selected according to production objectives. When quality optimization is the primary goal, T3 treatment (N-P_2_O_5_-K_2_O: 150-225-165 kg·ha^−1^, combined with an irrigation volume of 2400 m^3^·ha^−1^) is recommended.

## Data Availability

The original contributions presented in the study are included in the article/supplementary material. Further inquiries can be directed to the corresponding author.
